# Potential therapeutic impact of CD13 expression in non-small cell lung cancer

**DOI:** 10.1371/journal.pone.0177146

**Published:** 2017-06-12

**Authors:** Lars Henning Schmidt, Caroline Brand, Janine Stucke-Ring, Christoph Schliemann, Torsten Kessler, Saliha Harrach, Michael Mohr, Dennis Görlich, Alessandro Marra, Ludger Hillejan, Carsten Müller-Tidow, Georg Lenz, Eva Wardelmann, Rainer Wiewrodt, Wolfgang E. Berdel, Christian Schwöppe, Wolfgang Hartmann

**Affiliations:** 1 Department of Medicine A, Hematology, Oncology and Respiratory Medicine, University Hospital Muenster, Muenster, Germany; 2 Institute of Biostatistics and Clinical Research, University of Muenster, Muenster, Germany; 3 Department of Thoracic Surgery, Klinikum Bremen-Ost, Bremen, Germany; 4 Department of Thoracic Surgery, Niels-Stensen-Kliniken, Ostercappeln, Germany; 5 Department of Medicine IV, Hematology and Oncology, University Hospital of Halle (Saale), Halle (Saale), Germany; 6 Translational Oncology, University Hospital Münster, Münster, Germany; 7 Cluster of Excellence EXC 1003, Cells in Motion, Münster, Germany; 8 Gerhard-Domagk-Institute of Pathology, University of Muenster, Muenster, Germany; Technische Universitat Dresden, GERMANY

## Abstract

**Background:**

Aminopeptidase N (CD13) is a zinc-binding protease that has functional effects on both cancerogenesis and tumor angiogenesis. Since CD13 is an antigen suitable for molecular targeted therapies (e.g. tTF-NGR induced tumor vascular infarction), we evaluated its impact in NSCLC patients, and tested the effects of the CD13-targeted fusion protein tTF-NGR (truncated tissue factor (tTF) containing the NGR motif: asparagine-glycine-arginine) *in vivo* in nude mice.

**Methods:**

Expression of both CD13 and CD31 was studied in 270 NSCLC patients by immunohistochemistry. Clinical correlations and prognostic effects of the expression profiles were analyzed using univariate and multivariate analyses. In addition, a microarray-based analysis on the basis of the KM plotter database was performed. The *in vivo* effects of the CD13-targeted fusion protein tTF-NGR on tumor growth were tested in CD1 nude mice carrying A549 lung carcinoma xenotransplants.

**Results:**

CD13 expression in tumor endothelial and vessel associated stromal cells was found in 15% of the investigated samples, while expression in tumor cells was observed in 7%. Although no significant prognostic impact was observed in the full NSCLC study cohort, both univariate and multivariate models identified vascular CD13 protein expression to correlate with poor overall survival in stage III and pN2+ NSCLC patients. Microarray-based mRNA analysis for either adenocarcinomas or squamous cell carcinomas did not reveal any significant effect. However, the analysis of CD13 mRNA expression for all lung cancer histologies demonstrated a positive prognostic effect. *In vivo*, systemic application of CD13-targeted tissue factor tTF-NGR significantly reduced CD13+ A549 tumor growth in nude mice.

**Conclusions:**

Our results contribute a data basis for prioritizing clinical testing of tTF-NGR and other antitumor molecules targeted by NGR-peptides in NSCLC. Because CD13 expression in NSCLC tissues was found only in a specific subset of NSCLC patients, rigorous pre-therapeutic testing will help to select patients for these studies.

## Introduction

Lung cancer is still the leading cause of cancer-related death world-wide [1]. The two major histological subgroups are non-small cell lung cancer (NSCLC) representing ~85% and small cell lung cancer (SCLC) making up ~ 15% of the cases [2]. The majority of patients are diagnosed in advanced and often metastasized stages, leading to poor prognosis. To improve this situation both, better diagnostic and therapeutic strategies are needed.

Several cancer-related antigens are currently under clinical evaluation. Among these antigens, Aminopeptidase N (APN, CD13) is an interesting candidate for vascular targeting. CD13 is a membranous glycoprotein [3] with expression on endothelial cells, pericytes, and myeloid cells [4–6]. However, protein atlas shows a rather restricted presence of the protein in normal tissues (http://www.proteinatlas.org/ENSG00000166825-ANPEP/tissue). As a zinc-binding protease, Aminopeptidase N is responsible for extracellular proteolysis [7].

Increased CD13 expression levels are found in angiogenic endothelial cells [8]. Since angiogenesis is a major condition for cancer growth, high CD13 expression levels are also found in various malignant tissues [9,10] including lung cancer [11]. Besides angiogenesis-related expression, a predominantly peritumoral expression is reported [11,12]. Several functional mechanisms are described for CD13; apart from its potential impact on angiogenesis, CD13 might also affect tumor progression, cellular survival [9], and susceptibility towards chemotherapy [13–15]. Consequently, CD13 can affect prognosis in various tumor subtypes [9,16–18].

The observations that CD13 is a major binding site for the asparagine–glycine–arginine (NGR) peptide motif, and it is predominantly expressed on angiogenic endothelial cells [8,12] qualifies this antigen for targeted delivery of distinct anti-cancer molecules [19]. At present, two NGR-coupled molecules are under study: (1) NGR-tumor necrosis factor (NGR-hTNF [20]) and (2) truncated tissue factor-NGR (tTF-NGR [21–24]). Our scientific interest focuses mainly on a targeted procoagulatory effect of tTF-NGR. Upon the intravenous infusion of tTF-NGR, we could demonstrate vascular infarction in tumor blood vessels with subsequent retardation of tumor growth [22].

Here, we evaluate CD13 expression and its prognostic impact in human NSCLC, as well as demonstrate *in vivo* therapeutic activity of tTF-NGR against CD13+ tumor NSCLC xenografts.

## Methods

### Study population

Clinical follow-up information and sufficient tumor material of 270 curatively resected NSCLC patients (212 NSCLC patients from the Thoracic Departments in Ostercappeln, Germany; 58 NSCLC patients from the University Hospital Mainz) were collected and examined. Patients with stage IV, neoadjuvant treatment, R1 or R2 resection status, or with non-specified tumor histology (e.g. NSCLC not otherwise specified) were excluded from our analysis. All tissue samples were embedded in tissue microarrays. The original patient database contains 304 patient samples. Due to loss of tissue sections from the arrays, immunohistochemical evaluation was not possible in 34 cases for CD13. Hence, 270 NSCLC patients were included for further evaluation. Approval of the study by the Ethical committees of the regional physicians´ chamber of Westfalia-Lippe and the University of Muenster and the University of Mainz were obtained for the collection of paraffin-embedded tissue samples for biomarker testing. Due to the retrospective, anonymous character of our study, written consent was not needed. Clinical TNM staging (including clinical examination, CT scans, sonography, endoscopy, MRI, bone scan) was based on IUCC/AJCC recommendations (7^th^ edition). In terms of definite tumor staging, pathological exploration was carried out post-surgically. Primary pulmonary lesions were pathologically classified based on the WHO 2004 guidelines; 123 specimens were determined to be squamous cell carcinoma, 107 were adenocarcinoma, and 40 were large cell carcinoma. Survival time was either computed from the date of histological diagnosis to death, or the date of last contact. Baseline information of the NSCLC population is shown in Table 1.

**Table 1:** Baseline characteristics of the NSCLC study population.

Parameter		% of non-missing values
**Median Age, years (Q1;Q3)**	66.4 (61.8; 72.1)	
**Male Sex, N(%)**		(n = 270)
	210	78%
**Performance status**		(n = 258)
ECOG 0	48	19%
ECOG I	196	76%
ECOG >II	14	5%
**Tumor stage**		(n = 267)
Stage I	182	68%
Stage II	58	22%
Stage III	27	10%
**Lymph nodes status**		(n = 270)
pN0	198	73%
pN1	55	20%
pN2	17	6%
**Tumor histology**		(n = 270)
Squamous cell carcinoma	123	46%
Adenocarcinoma	107	40%
Large cell carcinoma	40	15%
**Tumor grading**		(n = 265)
Low grade (G1)	6	2%
Intermediate grade (G2)	91	34%
High grade (G3)	168	63%
**Expression of CD 13 (immunohistochemistry)**		(n = 270)
in stromal cells	41	15%
in tumor cells	20	7%

### Meta-analysis of the prognostic value of CD13

To study the prognostic value of CD13 (202888_at), “The Kaplan-Meier plotter” (KM plotter) database (www.kmplot.com) was applied. This online survival analysis software facilitates the prognostic meta-analysis of biomarkers. The original catalog contains 2437 lung cancer patients and is based on the Gene Expression Omnibus (GEO– http://www.ncbi.nlm.nih.gov/geo/) database. For the evaluation of CD13, expression data of 1926 lung cancer patients were available. Further information regarding the prognostic value of biomarkers using transcriptomic data in NSCLC are described by Györffy *et al*. [25].

### Immunohistochemistry

For immunohistochemical staining, paraffin-embedded tumor tissues were applied. Of interest, for sufficient topic correlation of CD13 expression, CD31 staining was performed, too. After deparaffinization, tissue slides were steam heated for 16 minutes at 95°C in EDTA buffers (pH 8) for antigen retrieval, followed by pretreatment with peroxidase inhibitor and the subsequent immunostaining with a 24 min incubation period of 50 μl CD13 antibody (clone: 38C12, mouse monoclonal antibody, Leica Biosystems, Buffalo Grove, USA) and of 50 μl CD31 antibody (clone: JC70, mouse monoclonal antibody, Cell Marque, Rocklin, Ca, USA). Immunoreaction was visualized with a biotinylated secondary antibody (OptiView DAB IHC Detection Kit, Ventana Medical Systems, Inc., Tucson, USA), according to the manufacturer. Finally, tissue microarrays (TMA) were counterstained with hematoxylin and covered with Cytoseal (Thermo Fisher Scientific, Inc., Waltham, USA).

Upon histological examination, immunohistochemical expression was evaluated analogous to the “Scoring HercepTest^™^—Breast” guidelines for HER2 protein over-expression assessment [26]. Here, four scores are differentiated: 0 and 1+ = negative expression; 2+ = weak expression; 3+ = strongly positive expression. Of interest, for statistical investigation a dichotomized evaluation system was performed (0/1+ *vs*. 2+/3+). The immunohistochemical evaluation of the TMA slides was performed by two independent investigators.

### Immunofluorescence staining

For immunofluorescence, 4 μm paraffin sections were deparaffinized, and tissue slides were steam heated for 30 minutes at 95°C (citrate buffer pH 6.1) for antigen retrieval followed by a 10% BSA blocking step. Sections were incubated over night at 4°C with a CD13 antibody (SP187, Cell Marque; dilution 1:300) and a CD31 antibody (JC70A, DAKO, dilution 1:200) followed by a 60 min incubation with a Cy3-labelled goat anti-rabbit-IgG antibody (#111-165-003, Dianova; dilution 1:200) and a FITC-labelled goat anti-mouse-IgG (#554001, BD Pharmingen; dilution 1:500). Appropriate PBS washing steps were employed, nuclei were counterstained with DAPI using a standard procedure. Since the antibodies used for CD13 and CD31 staining are species-specific for human CD13 and CD31, vascular and perivascular staining were not assayed in the xenografts.

### Generation of tTF-NGR

The full protocol for cloning, expression, and purification of tTF-NGR was published before [21–23].

### Tumor xenograft model

All procedures on animals were performed in agreement with German regulations (*i*.*e*. Tierversuchsgesetz §8, Abs. 2) and specifically approved by a project license (reference number: 84–02.04.2012.A247; licence provided by Landesamt für Natur, Umwelt und Verbraucherschutz Nordrhein-Westfalen (LANUV), Recklinghausen, Germany). CD-1 nude mice were purchased from Charles River Laboratories (Charles River Laboratories, Sulzfeld, Germany) and acclimated to our animal-experiment facility for at least 1 week before initiation of experiments. Mice were maintained in individually-ventilated cages (IVC) on a 12:12h light:dark cycle in a low-stress environment (22°C, 50% humidity, low noise) and given food and water *ad libitum*.

For *in vivo* tests, the human A549-lung carcinoma cell line [27] was purchased from ATCC (CCL-185; Manassas, VA, USA) and cultured in Ham´s F12 medium supplemented with 10% fetal calf serum and 1 mM glutamine at 37°C, high humidity, and 5% CO_2_. Cell viability was evaluated by trypan blue exclusion assay. A549 is an epithelial cell line derived from a patient suffering from lung carcinoma. Cell line identity was authenticated and confirmed by short tandem repeat (STR) profiling before and after experiments. Single tumor cell suspensions were injected subcutaneously (s.c.) into the right anterior flank of female CD-1 nude mice (9–12 weeks old). For therapeutic experiments, tumor growth was allowed to a mean volume as indicated in the Results section. Mice were then randomly assigned to receive either tTF-NGR or control saline intravenously (i.v.). Tumor size was evaluated using a standard caliper measuring tumor length and width in a blinded fashion, and the tumor volume was calculated using the formula: length x width x 0.52. Animals were killed by cervical dislocation in deep CO_2_ anesthesia, and primary tumors were surgically removed and measured. Tumor tissue was embedded in paraffin, sliced in 10 μm thick sections and stained with Haematoxylin-Eosin (H&E) and with a monoclonal CD13 antibody as described above.

### Flow cytometry

CD13 expression of A549 cells was analyzed by flow cytometry using the BD FACS Calibur flow cytometer (Becton-Dickinson (BD), San Jose, CA, USA). Briefly, 90% confluent cells were trypsinized (10% Trypsin, Gibco, Eggenstein, Germany), washed twice with PBS and blocked with human immunoglobulin G (IgG; 1 μg/1 x 10^5^ cells). For direct staining of the cell surface protein, cells were incubated with the monoclonal mouse anti-CD13-phycoerythrin (PE) antibody (ab69775, Abcam; 2 μl/1 x 10^5^ cells) for 30 minutes at 4°C. After two washing steps with ice-cold PBS/10% FCS, cells were resuspended in 500 μl PBS/FCS and analyzed in the flow cytometer.

### Statistical analysis

SPSS (SPSS Statistics, Version 22.0 released 2013, IBM Corp., Armonk, USA) was used for all statistical analyses. Of interest, data collection was performed retrospectively.

The study population was described by standard descriptive statistical measures. For categorical variables, absolute and relative frequencies are reported. Continuous variables are described by mean, standard deviation, median and inter-quartile range (IQR). Survival time is defined from first diagnosis until death. Univariate overall survival analysis was performed using the Kaplan-Meier method and log rank tests. A multivariable Cox proportional hazards model was fitted using a forward step-wise variable selection (inclusion criteria: p-value of the likelihood ratio test ≤ 0.05) to identify independent prognostic factors for overall survival. We considered potential prognostic factors that are tolerably complete (less than ten missing values, and with at least ten cases) to prevent statistical problems emerging from low sample size and extreme values. Patients with missing values in the cofactors were excluded from the analysis. Inferential statistics are intended to be exploratory (hypotheses generating), not confirmatory, and are interpreted accordingly. The comparison-wise type-I error rate is controlled instead of the experiment-wise error rate. The local significance level is set to 0.05. No adjustment for multiple testing is performed. Therefore, an overall significance level is not determined and cannot be calculated.

## Results

### Expression of CD13 and CD31 in NSCLC tissues

**Fig 1:**
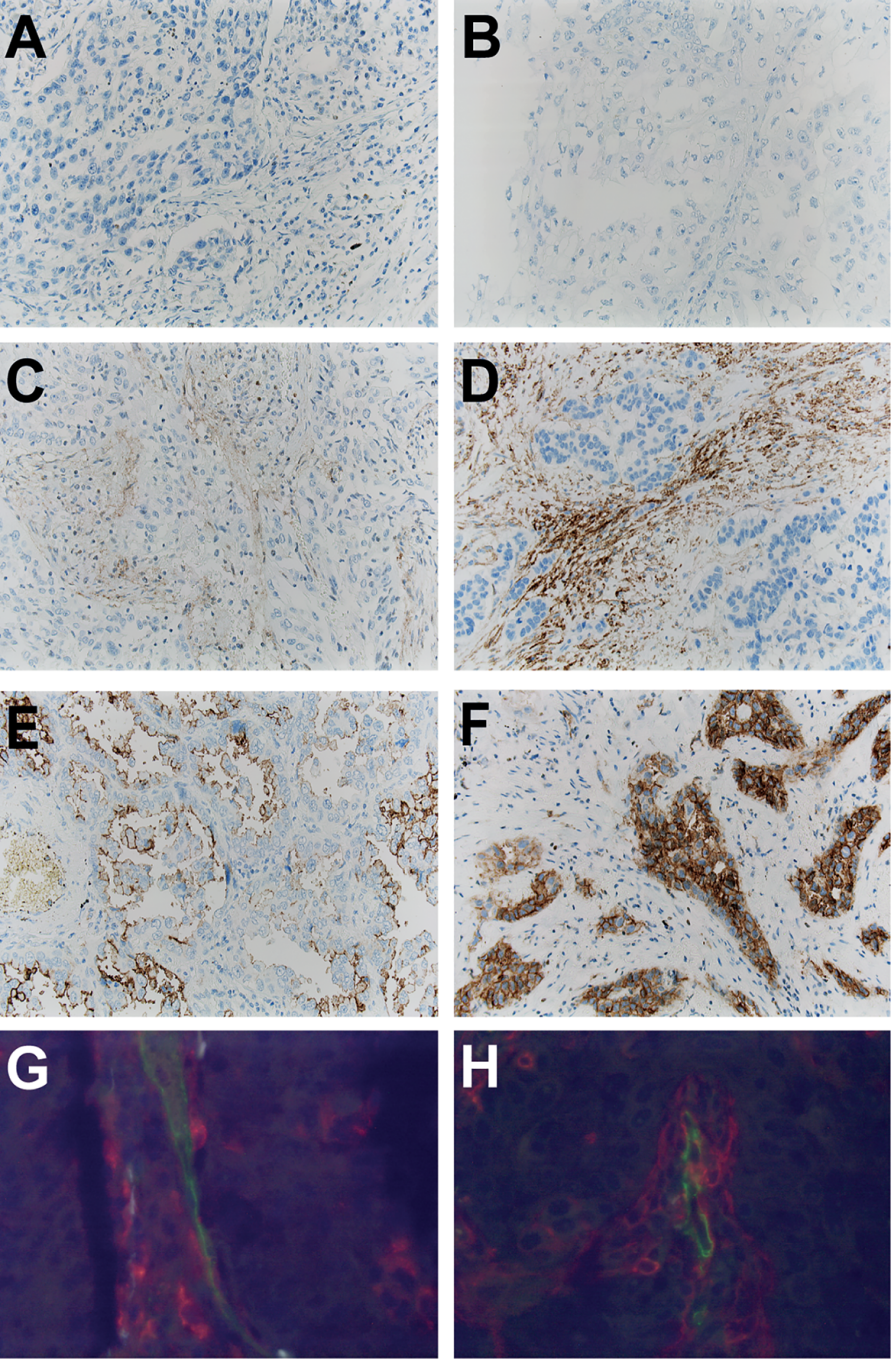
Expression of CD13 in NSCLC tissues. Examples for negative immunostaining are shown for squamous cell carcinoma (**Fig. 1A)** and for adenocarcinoma (**Fig. 1B**). Positive staining results are given for squamous cell carcinomas (stromal expression of CD13) (**Fig. 1C and Fig. 1D**) and for adenocarcinomas (tumor cell expression of CD13) (**Fig. 1E and Fig. 1F**). Co-staining of CD31 (green) and of CD13 (red) demonstrates CD13 expression in endothelial cells and perivascular stroma cells in a case of adenocarcinoma (**Fig. 1G)** and squamous cell carcinoma (**Fig. 1H**).

Baseline characteristics of n = 270 NSCLC patients with available immunohistochemical information for CD13 are summarized in Table 1. A positive immunostaining (*i*.*e*. 2+/3+, according to the criteria described above) of tumor tissues with the monoclonal CD13 antibody was found for endothelial cells and vessel-associated stroma cells in n = 41 cases (15%) and in NSCLC tumor cells in n = 20 cases (7%), the latter with a predominantly membranous immunostaining (Fig 1A–1F). As expected, endothelial cells of the investigated NSCLC tumor tissues displayed a positive expression for CD31 (S1 Fig). Using immunofluorescence staining, co-expression of CD13 and CD31 was also investigated (Fig 1G and 1H).

### Correlation of CD13 immunostaining with clinical parameters

With regard to clinicopathological parameters, positive correlations for CD13 expression in tumor cells (*i*.*e*. 2+/3+) were found both for adenocarcinoma tumor histology (p = 0.007; chi-squared test) and low tumor grading (p = 0.030; chi-square test). For CD13 expression in endothelial cells and vessel-associated stroma cells, positive correlations were not found for sex, tumor stage, lymph node status, tumor histology, nor for tumor grading (chi-square test for all: p > 0.05; Table 2).

**Table 2:** Correlations of clinicopathological variables with CD13 in NSCLC patients.

Clinical subgroups	Immunohistochemical expression of CD13	
	stromal cells	tumor cells
	p value*	p value*
**Sex**	*0*.*422*	*0*.*166*
Male	30/41	13/20
Female	11/41	7/20
**Tumor stage**	*0*.*282*	*1*.*000*
Stage I	25/41	14/20
Stage II-III	16/41	6/20
**Lymph nodes status**	*0*.*446*	*1*.*000*
pN0	28/41	15/20
pN1-3	13/41	5/20
**Tumor histology**		
	*0*.*866*	*0*.*019*
Squamous cell carcinoma	18/41	4/20
Non-squamous cell carcinoma	23/41	16/20
	*0*.*491*	*0*.*007*
Adenocarcinoma	14/41	14/20
Non adenocarcinoma	27/41	6/20
	*0*.*160*	*0*.*748*
Large cell carcinoma	9/41	2/20
Non large cell carcinoma	32/41	18/20
**Tumor grading**	*1*.*000*	*0*.*030*
Low and intermediate grade (G1 and G2)	15/40	12/20
High grade (G3)	25/40	8/20

n.e. = not evaluable;

*p values according to Fisher’s exact test

### Univariate prognostic effects of CD13 protein presence

**Fig 2:**
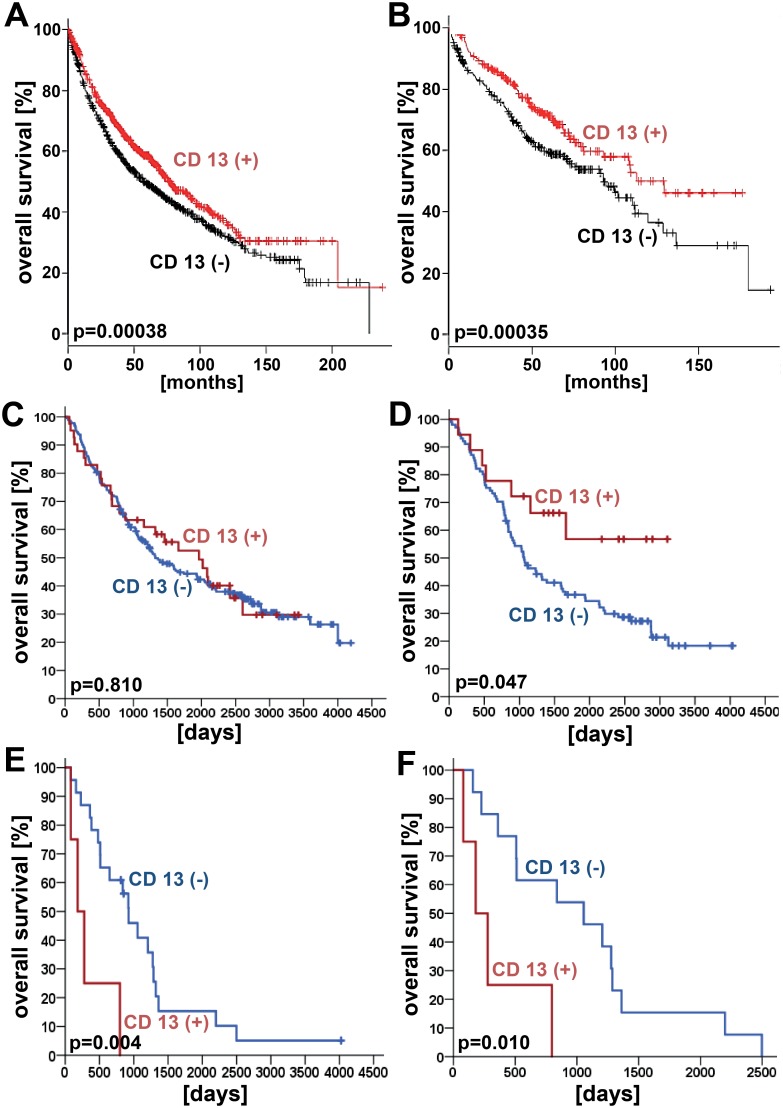
Prognostic impact of CD13 expression in non-small cell lung cancer (NSCLC). Univariate prognostic models (i.e. microarray-based mRNA expression) are shown for CD13 of all patients (**Fig. 2A**) and for stage I tumor patients (**Fig. 2B**) according to “The Kaplan-Meier plotter” database (www.kmplot.com [25]). With regard to the immuno-histochemical protein expression of CD13 in endothelial cells and vessel-associated stroma cells of the tumors prognostic analyses were performed for our complete NSCLC study collective (**Fig. 2C**), for squamous cell carinoma patients (**Fig. 2D**), for stage III NSCLC tumor patients (**Fig. 2E**), and for patients with pN2 lymph node status (**Fig. 2F**).

In the entire NSCLC study collective, neither endothelial cell or vessel-associated stroma cell expression, nor tumor cell expression of CD13 had a prognostic effect (both analyses p > 0.05; log rank test). However, depending on tumor histology, a marginal positive prognostic effect was found for endothelial cell and vessel-associated stroma cell CD13 expression for NSCLC patients with squamous cell carcinoma histology (n = 123 patients; p = 0.047, log rank test). Of interest, while examining the entire patient collective, a poor prognostic impact of endothelial cell and vessel-associated stroma cell CD13 expression on overall survival was detected for tumor stage III patients (n = 27 patients; p = 0.004, log rank test), and for patients with pN2 lymph node status (n = 17 patients; p = 0.010, log rank test). For all other tested clinical variables, no prognostic effects were found (p > 0.05; log rank test; Table 3). To visualize prognostic effects, Kaplan-Meier curves were generated (Fig 2A–2F).

**Table 3:** Univariate log rank test results for the association of CD13 with overall survival for the investigated study collective of n = 270 tissue microarray-embedded NSCLC patients (immunohistochemical analysis).

p-values* for	Immunohistochemical expression of CD13 in	
	stroma cells	tumor cells
**All patients**	0.810	0.702
**Histology**		
Squamous cell carcinoma	0.047	0.456
Adenocarcinoma	0.321	0.491
**Tumor stage**		
Tumor stage I	0.809	0.650
Tumor stage II	0.402	0.836
Tumor stage III	0.004	0.327
**Grading**		
Low grade (G1)	0.953	0.953
Intermediate grade (G2)	0.380	0.999
High grade (G3)	0.716	0.985
**Lymph node status**		
pN 0	0.819	0.775
pN 1	0.326	0.929
pN 2	0.010	n.e.

To compare our data to other available expression data for CD13, univariate prognostic models were performed on the basis of Affymetrix microarray data as provided by the “The Kaplan-Meier plotter” database (www.kmplot.com). In the full study lung cancer database (n = 1926 patients), CD13 mRNA expression was shown to be associated with increased overall survival (p = 0.00038; HR = 0.8 (95%-CI: 0.7–0.9)). In subgroup analyses lung cancer patients in tumor stage I (n = 577 patients) had an increased overall survival in case CD13 mRNA expression was observed (p = 0.0035; HR = 0.67 (95%-CI: 0.51–0.88)). For all other tested clinical variables including tumor histology, tumor grading, and lymph node status, no prognostic effects were found (Table 4).

**Table 4:** Univariate log rank test results for the association of CD13 with overall survival for the study collective of n = 1926 lung cancer patients as provided by “The Kaplan-Meier plotter” (KM plotter) database (transcriptomic data).

p-values*for	CD13 microarray expression
**All patients**	0.00038
**Histology**	
Squamous cell carcinoma	0.210
Adenocarcinoma	0.160
**Tumor stage**	
Tumor stage I	0.0035
Tumor stage II	0.500
Tumor stage III	0.510
**Grading**	
Low grade (G1)	0.410
Intermediate grade (G2)	0.950
High grade (G3)	0.900
**Lymph node status**	
pN 0	0.590
pN 1	0.330
pN 2	0.940

n.e. = not evaluable;

*p values according to log rank test

### Multivariate prognostic effects of CD13 protein expression

To identify independent prognostic factors, a multivariate Cox proportional hazard model was performed. All variables that were tested before in the univariate model were included (i.e. CD13 expression in endothelial cells and vessel-associated stroma cells, CD13 expression in NSCLC tumor cells, tumor stage, lymph node status and tumor grading). In the multivariate model for all investigated NSCLC patients, neither CD13 expression on endothelial cell and vessel-associated stroma cells, nor tumor cell expression of CD13 was of prognostic relevance (p > 0.05, likelihood ratio test). The only parameter with impact on overall survival was tumor stage (p < 0.001, likelihood ratio test; Table 5). Regarding clinical subgroups, the observed positive prognostic effect for endothelial cell and vessel-associated stroma cell expression of CD13 was not confirmed in the multivariate analysis (p > 0.05, likelihood ratio test; Table 5). However, the poor prognostic effect of endothelial cell and vessel-associated stroma cell expression of CD13 for stage III NSCLC patients (p = 0.009, HR = 4.956 (95%-CI: 1.486–16.526), likelihood ratio test; Table 5) and for NSCLC patients with pN2 lymph node status (p = 0.019, HR = 4.963 (95%-CI: 1.297–18.991) likelihood ratio test; Table 5) were confirmed.

**Table 5:** Overall survival: Exploratory prognostic factors in a Cox proportional hazards model. Included variables: CD13 expression in stromal cells (negative expression (ref.) *vs*. positive expression), CD13 expression in NSCLC tumor cells (negative expression (ref.) *vs*. positive expression), tumor stage (stage I (ref.) *vs*. stage II *vs*. stage III), lymph node status (pN0 (ref.) *vs*. pN1 *vs*. pN2 *vs*. pN2), and tumor histology (squamous cell carcinoma *vs*. adenocarcinoma *vs*. large cell carcinoma).

Prognostic factor	p-value	HR^1^(95% CI)^2^
**Multivariate analysis for all NSCLC patients**		
Tumor stage	<0.001	
Tumor stage I *vs*. II	0.001	1.842 (1.265–2.683)
Tumor stage I vs. III	<0.001	2.763 (1.735–4.398)
**Multivariate analysis for squamous cell carcinoma patients**		
Tumor stage	0.036	
Tumor stage I *vs*. II	0.026	1.784 (1.072–2.969)
Tumor stage I vs. III	0.071	2.012 (0.941–4.301)
**Multivariate analysis for stage III NSCLC patients**		
CD13 stromal cell expression	0.009	4.956 (1.486–16.526)
**Multivariate analysis for NSCLC patients with pN2 lymph node status**		
CD13 stromal cell expression	0.019	4.963 (1.297–18.991)

HR^1^ = Hazard ratio: HR <1 indicates improved survival;

CI^2^ = Confidence interval

### Therapeutic activity of tTF-NGR in the A549 tumor xenograft model

**Fig 3:**
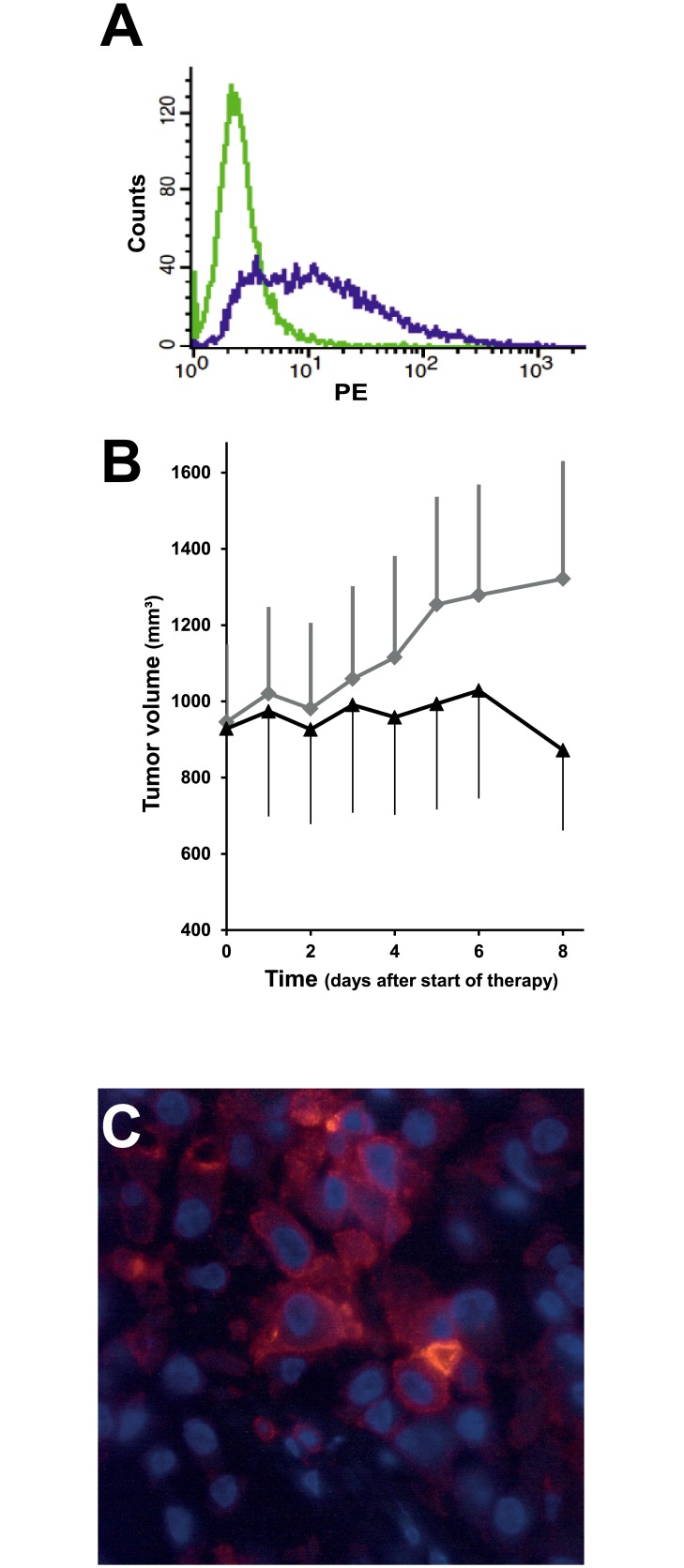
*In vivo* therapeutic activity of systemic tTF-NGR against CD13+ A549 tumor xenografts. To investigate CD13 expression flow cytometry was performed with a monoclonal PE-labeled anti-CD13 antibody. CD13 expression was found in 47% of the A549 lung cancer cells (green, control; purple, CD13) (**Fig. 3A**). Following treatment with tTF-NGR (1 mg tTF-NGR/kg x4 (arrows); i.v.; n = 4 CD-1 nude mice) tumor growth of subcutaneous A549 xenotransplants was reduced as compared to the saline control group (n = 6) CD-1 nude mice (**Fig. 3B)**. The CD13 expression in subcutaneous A549 xenotransplant is demonstrated by immunofluorescence; since the antibodies used for CD13 and CD31 staining are species-specific for human CD13 and CD31, vascular and perivascular staining were not assayed in the xenografts (**Fig. 3C**).

First, we studied expression of CD13 in A549 lung cancer cells. Using flow cytometry A549 lung cancer cells were identified to express CD13 in a fraction of 47% (Fig 3A). In the analysis of the therapeutic activity of tTF-NGR against A549 tumor xenografts, tumor growth was reduced by systemic i.v. application of tTF-NGR (n = 4 CD-1 nude mice) in comparison to the saline control group (n = 6 CD-1 nude mice; Fig 3B). Fig 3B shows one representative experiment out of multiple experiments, which overall showed statistically significant differences between control tumor growth versus tumor growth in tTF-NGR treated animals (p < 0.05, Mann-Whitney test).^22^ CD13 expression in tumor cells of the subcutaneous A549 xenotransplants was demonstrated by immunofluorescence staining (Fig 3C). Since the antibodies used for CD13 and CD31 staining are species-specific for human CD13 and CD31, vascular and perivascular staining were not assayed in the xenografts.

## Discussion

The zinc-binding protease Aminopeptidase N (APN, CD13) was shown to influence cancerogenesis and tumor angiogenesis [9]. For several tumor types, its prognostic effects have been reported [9,16,18,28]. Due to its potential impact on angiogenesis [9] and increased expression levels in endothelial cells and vessel-associated stroma cells [11,12], CD13 might be a preferable candidate for targeted therapies [19]. The therapeutic principle to inhibit CD13 activity was previously demonstrated for NSCLC patients by Ichinose *et al*. [29]. According to their analysis, postoperative survival rates of resected NSCLC patients with squamous cell carcinoma histology were improved after postoperative application of an oral CD13 inhibitor [29].

Our data contribute to the therapeutic principle of targeted therapies against CD13. For the application of the retargeted procoagulatory tissue factor tTF-NGR [21–24] we could already demonstrate vascular infarction of tumor blood vessels and subsequent tumor growth retardation [22]. Likewise, for subcutaneous CD13+ A549 xenotransplants in CD-1 nude mice similar results were found with a significant reduction of tumor growth after application of tTF-NGR in comparison to the saline control group, which reproduces earlier results (22). The fact, that tumor cells in addition to endothelial cells constitute parts of the inner cell layer of immature tumor neo-vessels [30] adds to the explanation of tumor vascular infarction induced by CD13 retargeted tTF in CD13+ tumors.

Against this background, we evaluated both the expression and the prognostic impact of CD13 in NSCLC patients. In addition, CD31 staining was performed both to visualize the vascular morphology and to detect discrete capillaries. Based on the visualization of the vascular structures, topic correlation of CD13 expression was possible. Since CD13 is predominantly expressed by fibroblasts around the blood vessels and endothelial cells next to tumor cells [31], CD13 expression was assessed separately both for endothelial cells and vessel-associated stroma cells, as well as for tumor cells. Overall, positive CD13 expression was found more often in tumor stroma (15% of the investigated patients) compared to tumor cells (7% of the investigated patients). This observation is in agreement with the data reported by Ito *et al*. [11] (32.6% in stromal cells *vs*. 9.5% in tumor cells). Of interest, in our study population CD13 expression in tumor cells was mainly restricted to tumors with low tumor grading and with adenocarcinoma histology. The latter observation was also reported for adenocarcinoma cell lines [16] and for adenocarcinoma NSCLC patients [11,15]. However, in contrast to Ito *et al*. [11], we did not find any association for CD13 expression in stromal cells and squamous cell carcinoma histology. Moreover, no relevant correlation was found in our analysis for lymph node status or tumor stage, as described before by Zhang *et al*. [15]. Discrepancies between our findings and those published by other study groups might be due to both, heterogeneity in patient cohorts or methodology (e.g. differing antibodies). Moreover, interobserver reliability in evaluating immunohistochemical studies still remains controversial and serves as a potential reason for discrepancies besides differing antibodies.

Prognostic impact of CD13 expression was investigated focusing on overall survival. For the complete cohort of NSCLC patients both univariate and multivariate survival analysis did not yield any relevant prognostic impact for CD13 expression in endothelial cells and vessel-associated stroma cells or in tumor cells (all analyses p > 0.05), which corresponds well to the study results reported by Ito *et al*. [11]. However, for distinct clinical subgroups prognostic effects were identified. On the one hand, the univariate model revealed a positive prognostic effect of borderline significance for CD13 expression in endothelial cells and vessel-associated stroma cells (p = 0.047; log rank test) for patients with squamous cell carcinoma histology. However, this prognostic impact was not confirmed by the multivariate model (p > 0.05, likelihood ratio test). On the other hand, both for stage III NSCLC patients and for NSCLC patients with pN2 lymph node status, CD13 expression in endothelial cells and vessel-associated stroma cells was identified to be associated with reduced overall survival (p < 0.05 both analyses, log rank test). Within the clinical subgroups, these unfavorable effects were both confirmed in the multivariate model (p < 0.05 both analyses, likelihood ratio test).

In summary, our study results suggest the poor prognostic effect of CD13 protein expression depends on tumor stage and lymph node status. Although other study groups did not offer a stratified analysis, they also contributed to the idea of a poor prognostic impact of CD13 expression in NSCLC [15,16] (S1 Table) as well as in other tumor types such as pancreatic carcinoma [28], ovarian cancer [18] and colon carcinoma [9]. This negative prognostic impact may be due to proteolysis of extracellular matrix components by CD13, supporting invasive capacity of endothelial cells in tumor neo-vessels or invasive capacity of CD13+ tumor cells.

In contrast to the discovered unfavorable effects of CD13 protein presence in a subgroup of NSCLC, the analysis of CD13 mRNA expression for all lung cancer patients (p = 0.00038; HR = 0.8; n = 1926 patients; all tumor histologies) on the basis of Affymetrix microarray data demonstrated a positive prognostic effect. Since, the histology stratified analysis for CD13 did not show any prognostic effect (p value for both analyses > 0.05) for squamous cell carcinoma patients (SCC: n = 524 patients) or for adenocarcinoma patients (ACA: n = 720 patients), part of the observed positive prognostic effect of CD13 might be related to other histological subtypes than SCC or ACA, although rare in numbers. An alternative explanation comparing the contradictory transcriptome data with our protein expression data could be variation in posttranscriptional regulation of CD13 biosynthesis. However, findings of RNA expression seem to be of less importance for the targeting approach discussed here.

In conclusion, CD13 expression by tumor endothelial cells and vessel-associated stroma cells as well as in tumor cells of patients suffering from advanced NSCLC, could constitute an interesting target for a small subgroup of NSCLC patients. As one example of CD13-targeted experimental therapy tTF-NGR could be further studied. Due to the fact that CD13 expression in NSCLC tissues was found only in a restricted subset of NSCLC patients rigorous pretherapeutic staining should help to select patients for this treatment approach. However, as an approach to a more individualized treatment of NSCLC patients, a subgroup representing 15% of the patients justifies further studies.

## Supporting information

S1 Fig Representative example for CD31 expression in NSCLC microvessels. (EPS)

S2 Fig FACS analysis (A 549; raw data). (PDF)

S1 Table The prognostic impact of CD13 in lung cancer with regard to univariate analyses. (PDF)

S2 Table Mouse trial: A549 Xenotransplants (tTF-NGR *vs*. PBS; raw data). (PDF)

S3 Table NSCLC study collective (CD13 expression; raw data). (PDF)
