# Correction: Potential therapeutic impact of CD13 expression in non-small cell lung cancer

**DOI:** 10.1371/journal.pone.0183201

**Published:** 2017-08-09

**Authors:** Lars Henning Schmidt, Caroline Brand, Janine Stucke-Ring, Christoph Schliemann, Torsten Kessler, Saliha Harrach, Michael Mohr, Dennis Görlich, Alessandro Marra, Ludger Hillejan, Carsten Müller-Tidow, Georg Lenz, Eva Wardelmann, Rainer Wiewrodt, Wolfgang E. Berdel, Christian Schwöppe, Wolfgang Hartmann

Fig 3 appears incorrectly. Please see the complete, correct Fig 3 here.

**Fig 3:**
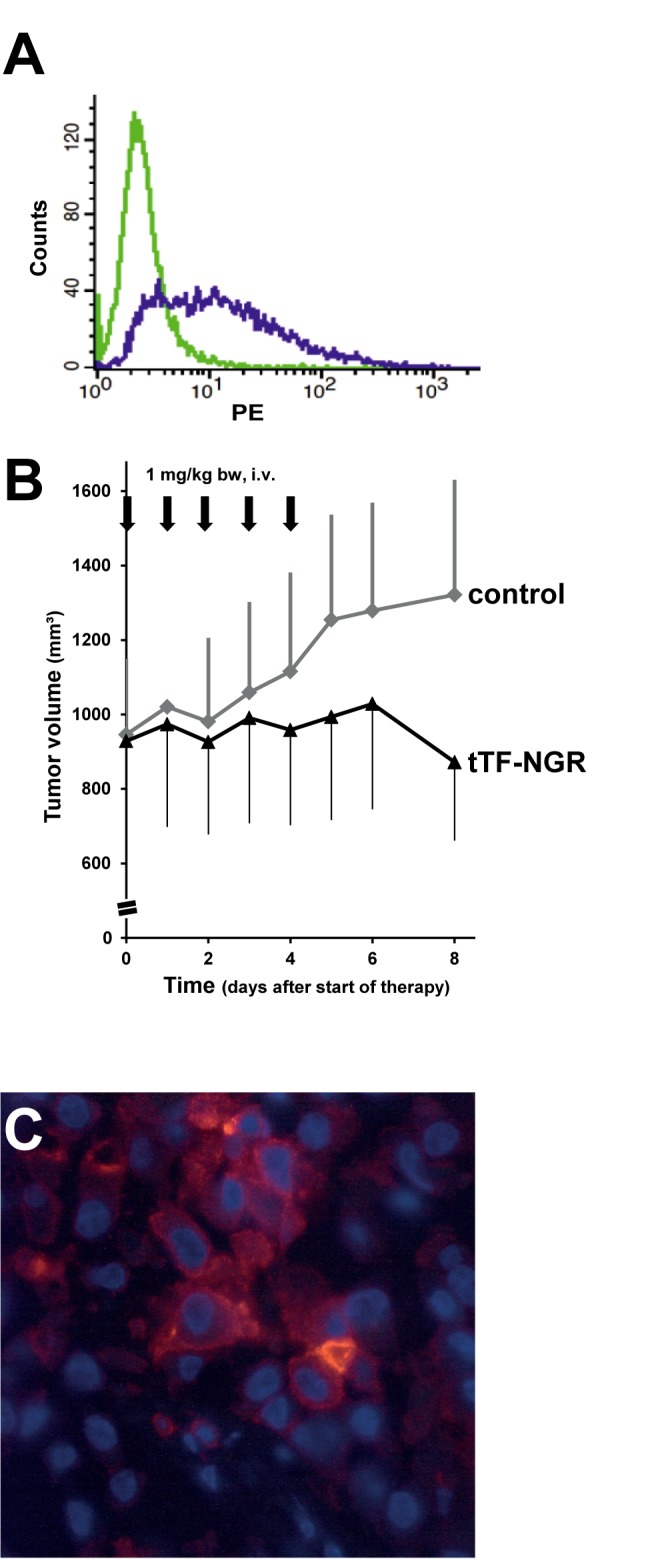
In vivo therapeutic activity of systemic tTF-NGR against CD13+ A549 tumor xenografts. To investigate CD13 expression flow cytometry was performed with a monoclonal PE-labeled anti-CD13 antibody. CD13 expression was found in 47% of the A549 lung cancer cells (green, control; purple, CD13) (Fig 3A). Following treatment with tTF-NGR (1 mg tTF-NGR/kg x5 (arrows); i.v.; n = 4 CD-1 nude mice) tumor growth of subcutaneous A549 xenotransplants was reduced as compared to the saline control group (n = 6) CD-1 nude mice (Fig 3B). The CD13 expression in subcutaneous A549 xenotransplant is demonstrated by immunofluorescence; since the antibodies used for CD13 and CD31 staining are speciesspecific for human CD13 and CD31, vascular and perivascular staining were not assayed in the xenografts (Fig 3C).
