# Supplementary material for: Potential therapeutic impact of CD13 expression in non-small cell lung cancer
Source: PLoS One. 2017 Jun 12;12(6):e0177146. doi: 10.1371/journal.pone.0177146 (PMC5467809; doi:10.1371/journal.pone.0177146)
Supplement: S2 Fig — (PDF) [file pone.0177146.s002.pdf]

Raw data: **FACS A549**
anti-CD-13-PE

unstained cells

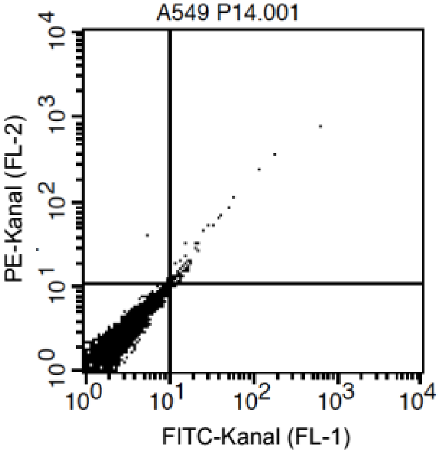

Isotype control (PE)

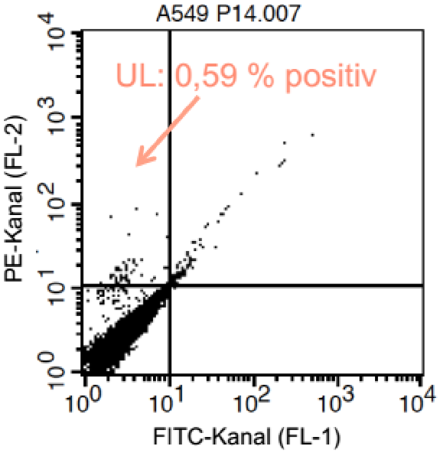

m-anti-CD13 (PE)

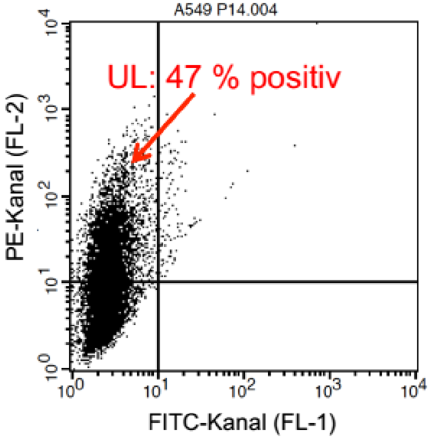

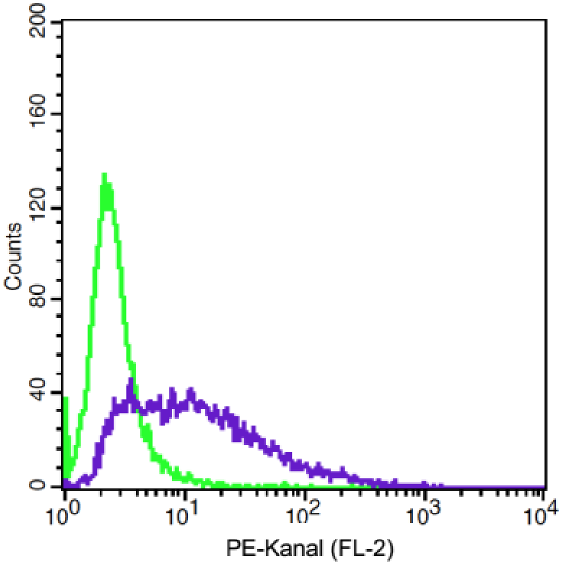

unstained cells
m-anti-CD13 (PE) stained cells
