# Supplementary material for: Potential therapeutic impact of CD13 expression in non-small cell lung cancer
Source: PLoS One. 2017 Jun 12;12(6):e0177146. doi: 10.1371/journal.pone.0177146 (PMC5467809; doi:10.1371/journal.pone.0177146)
Supplement: S1 Table — (PDF) [file pone.0177146.s003.pdf]

S1 Table. The prognostic impact of CD13 in lung cancer with regard to univariate analyses.

**Author**	**Study patients (n)**	**Mab Clone**	**Study population**	**Prognostic value of CD 13**	**Log rank test p-value**
**Tokuhara *et al.* 2006**[16]	194	MH8-11	n = 194 NSCLC patients	poor	0.006
			n = 122 patients with ACA[#1]	poor	0.0172
			n = 63 patients with SCC[#2]	poor	0.0198
**Ito *et al.* 2009**[11]	95	13C03 [IHC#3]	n = 95 NSCLC patients	c.n.s.[#5]	0.598
			n = 50 patients with ACA[#1]	c.n.s.[#5]	0.983
			n = 34 patients with SCC[#2]	c.n.s.[#5]	0.241
		13C03 [IHC#4]	n = 95 NSCLC patients	c.n.s.[#5]	0.518
			n = 50 patients with ACA[#1]	c.n.s.[#5]	---
			n = 34 patients with SCC[#2]	c.n.s.[#5]	0.360
**Zhang *et al.* 2015**[15]	127	38C12	n = 58 patients with ACA[#1]	poor	0.016
			n = 69 patients with SCC[#2]	poor	0.001
**Schmidt *et al.* 2017**	270	38C12 [IHC#3]	n = 270 NSCLC patients	c.n.s.[#5]	0.810
			n = 107 patients with ACA[#1]	c.n.s.[#5]	0.321
			n = 123 patients with SCC[#2]	borderline good	0.047
		38C12 [IHC#4]	n = 270 NSCLC patients	c.n.s.[#5]	0.702
			n = 107 patients with ACA[#1]	c.n.s.[#5]	0.491
			n = 123 patients with SCC[#2]	c.n.s.[#5]	0.456

[#1] ACA = Adenocarcinoma
[#2] SCC = Squamous cell carcinoma
[#3] IHC = Immunohistochemical expression of CD13 in stroma cells
[#4] IHC = Immunohistochemical expression of CD13 in tumor cells
[#5] c.n.s. = Clinically not significant
