# Supplementary material for: Potential therapeutic impact of CD13 expression in non-small cell lung cancer
Source: PLoS One. 2017 Jun 12;12(6):e0177146. doi: 10.1371/journal.pone.0177146 (PMC5467809; doi:10.1371/journal.pone.0177146)
Supplement: S2 Table — (PDF) [file pone.0177146.s004.pdf]

# Raw data: Mouse trial

## A549 Xenotransplants

## tTF-NGR *vs.* PBS

| A | B | C | D | E | F | G | H | I | J | K | L | M | N | O | P | Q |
|---|---|---|---|---|---|---|---|---|---|---|---|---|---|---|---|---|
| Day | | | | 0 | | | 1 | | | 2 | | | 3 | | | 4 |
| Therapy | | | Therapy | | | Therapy | | | Therapy | | | Therapy | | | Therapy | |
| | weight | length | width | volume | length | width | volume | length | width | volume | length | width | volume | length | width | volume |
| **PBS** | 39 | 17,6 | 14 | 1793,792 | 18,4 | 14,4 | 1984,02048 | 18,8 | 14 | 1916,096 | 19,1 | 14,5 | 2088,203 | 19,8 | 14,9 | 2285,81496 |
| **PBS** | 35 | 13,4 | 9,8 | 669,20672 | 13,8 | 9,6 | 661,34016 | 13,4 | 9,6 | 642,17088 | 13,9 | 9,8 | 694,17712 | 14,3 | 10,2 | 773,64144 |
| **PBS** | 26 | 8,4 | 6,7 | 196,07952 | 7,8 | 6,7 | 182,07384 | 7,6 | 6,4 | 161,87392 | 8,6 | 6,8 | 206,78528 | 8,8 | 6,7 | 205,41664 |
| **PBS** | 34 | 12,8 | 10,4 | 719,91296 | 12,7 | 11,5 | 873,379 | 13,3 | 10,4 | 748,03456 | 13,2 | 10,5 | 756,756 | 13,5 | 10,7 | 803,7198 |
| **PBS** | 35 | 15,8 | 11,9 | 1163,46776 | 16,4 | 12,1 | 1248,58448 | 16,6 | 12,1 | 1263,81112 | 16,8 | 12,1 | 1279,03776 | 16,8 | 13 | 1476,384 |
| **PBS** | 30 | 16,2 | 11,6 | 1133,53344 | 16,5 | 11,7 | 1174,5162 | 16,5 | 11,6 | 1154,5248 | 16,1 | 12,6 | 1329,13872 | 16,7 | 11,5 | 1148,459 |
| | | | **203,5328863** | **945,9987** | | **227,1632979** | **1020,6524** | | **225,1260804** | **981,0852** | | **243,3200599** | **1059,0163** | | **265,7947475** | **1115,5726** |
| **TTF-NGR** | 36 | 8,3 | 7,2 | 223,74144 | 8,4 | 7,1 | 220,19088 | 8,7 | 7,4 | 247,73424 | 8,1 | 6,9 | 200,53332 | 8,3 | 7 | 211,484 |
| **TTF-NGR** | 35 | 14 | 10,4 | 787,4048 | 13,8 | 12,2 | 1068,07584 | 14,1 | 10,2 | 762,82128 | 14,8 | 12,5 | 1202,5 | 14,8 | 12,9 | 1280,69136 |
| **TTF-NGR** | 29 | 18,3 | 13,2 | 1658,06784 | 18,6 | 13,5 | 1762,722 | 18,4 | 13 | 1616,992 | 18,2 | 13,6 | 1750,46144 | 18 | 12,9 | 1557,5976 |
| **TTF-NGR** | 33 | 14,2 | 11,9 | 1045,64824 | 14,2 | 10,7 | 845,39416 | 14,4 | 12 | 1078,272 | 14,4 | 10,4 | 809,90208 | 14,5 | 10,2 | 784,4616 |
| | | | **257,7044384** | **928,7156** | | **275,6469971** | **974,0957** | | **248,4109013** | **926,4549** | | **282,7485485** | **990,8492** | | **256,3015146** | **958,5586** |

| | | | | | | | | | | |
|---|---|---|---|---|---|---|---|---|---|---|
| Day | | | 5 | | | 6 | | | | 8 |
| Therapy | | | | | | | | | | |
| | length | width | volume | length | width | volume | weight | length | width | volume |
| **PBS** | 20,2 | 15,3 | 2458,88136 | 20,2 | 15,5 | 2523,586 | 39 | 20,4 | 15,8 | 2648,18112 |
| **PBS** | 13,5 | 12 | 1010,88 | 14,3 | 10,4 | 804,27776 | 35 | 14,3 | 10,8 | 867,33504 |
| **PBS** | 8,5 | 7 | 216,58 | 9 | 7,4 | 256,2768 | 26 | 9,3 | 7,2 | 250,69824 |
| **PBS** | 14,4 | 10,7 | 857,30112 | 14,1 | 11,9 | 1038,28452 | 34 | 14,2 | 11,8 | 1028,14816 |
| **PBS** | 17,9 | 13 | 1573,052 | 17,7 | 13,3 | 1628,09556 | 35 | 17,7 | 14 | 1803,984 |
| **PBS** | 17,9 | 12,3 | 1408,20732 | 17,8 | 12,4 | 1423,20256 | 30 | 17,2 | 12,2 | 1331,22496 |
| | | **282,3527** | **1254,1503** | | **289,68** | **1278,9539** | | | **308,7567204** | **1321,5953** |
| **TTF-NGR** | 8,3 | 6,9 | 205,48476 | 8,1 | 7,1 | 212,32692 | 36 | 8,4 | 6,9 | 207,96048 |
| **TTF-NGR** | 15,3 | 12,8 | 1303,51104 | 15,4 | 13 | 1353,352 | 35 | 15,3 | 12,7 | 1283,22324 |
| **TTF-NGR** | 18 | 13,4 | 1680,6816 | 18,1 | 13,5 | 1715,337 | 29 | 14,3 | 12,6 | 1180,53936 |
| **TTF-NGR** | 14,8 | 10,1 | 785,06896 | 15,1 | 10,3 | 833,01868 | 33 | 14,8 | 10,3 | 816,46864 |
| | | **277,5717** | **993,6866** | | **283,022** | **1028,5087** | | | **210,4063474** | **872,0479** |
