# Supplementary material for: Potential therapeutic impact of CD13 expression in non-small cell lung cancer
Source: PLoS One. 2017 Jun 12;12(6):e0177146. doi: 10.1371/journal.pone.0177146 (PMC5467809; doi:10.1371/journal.pone.0177146)
Supplement: S3 Table — (PDF) [file pone.0177146.s005.pdf]

Raw data: NSCLC Study Collective; CD13 expression

#	CD13 Stroma	CD13 Tumor	Age	Sex	ECOG	Squamous cell carcinoma	Adenocarcinoma
1	negative	negative	87,6	male	ECOG 2	no SCC	no ACA
2	negative	negative	62,4	male	ECOG 2	no SCC	Adenocarcinoma
3	negative	negative	68,3	male	ECOG 2	no SCC	Adenocarcinoma
4	negative	positive	67,2	male	ECOG 2	Squamous cell carcinoma	no ACA
5	positive	negative	74,4	male	ECOG 2	Squamous cell carcinoma	no ACA
6	negative	negative	58,3	male	ECOG 2	Squamous cell carcinoma	no ACA
7	negative	negative	72,9	female	ECOG 2	Squamous cell carcinoma	no ACA
8	negative	negative	71,4	male	ECOG 2	Squamous cell carcinoma	no ACA
9	negative	negative	76,7	male	ECOG 2	Squamous cell carcinoma	no ACA
10	negative	negative	79	male	ECOG 2	no SCC	Adenocarcinoma
11	negative	negative	69,3	male	ECOG 2	Squamous cell carcinoma	no ACA
12	negative	negative	76,5	male	ECOG 2	no SCC	Adenocarcinoma
13	negative	negative	65,2	male	ECOG 2	Squamous cell carcinoma	no ACA
14	negative	negative	82,4	male	ECOG 2	Squamous cell carcinoma	no ACA
15	positive	positive	76,9	female	ECOG 1	no SCC	no ACA
16	negative	positive	73,7	male	ECOG 1	no SCC	no ACA
17	positive	negative	74,4	male	ECOG 1	no SCC	no ACA
18	positive	negative	66,4	female	ECOG 1	no SCC	no ACA
19	positive	negative	71,8	male	ECOG 1	no SCC	no ACA
20	negative	negative	69,6	male	ECOG 1	no SCC	no ACA
21	negative	negative	77,1	male	ECOG 1	no SCC	no ACA
22	negative	negative	72,6	female	ECOG 1	no SCC	no ACA
23	negative	negative	54,9	female	ECOG 1	no SCC	no ACA
24	negative	negative	61,8	female	ECOG 1	no SCC	no ACA
25	negative	negative	74,6	male	ECOG 1	no SCC	no ACA
26	negative	negative	67,3	male	ECOG 1	no SCC	no ACA
27	negative	negative	68,4	male	ECOG 1	no SCC	no ACA
28	negative	negative	64,7	male	ECOG 1	no SCC	no ACA
29	negative	negative	70,1	female	ECOG 1	no SCC	no ACA
30	negative	negative	66	male	ECOG 1	no SCC	no ACA
31	negative	negative	57,6	male	ECOG 1	no SCC	no ACA
32	negative	negative	70,9	male	ECOG 1	no SCC	no ACA
33	negative	negative	70,2	male	ECOG 1	no SCC	no ACA
34	negative	negative	66,4	male	ECOG 1	no SCC	no ACA
35	negative	negative	60,4	male	ECOG 1	no SCC	no ACA
36	negative	negative	68,7	male	ECOG 1	no SCC	no ACA
37	negative	negative	66,1	female	ECOG 1	no SCC	no ACA
38	positive	positive	51,8	male	ECOG 1	no SCC	Adenocarcinoma
39	negative	positive	70,5	male	ECOG 1	no SCC	Adenocarcinoma
40	negative	positive	57,5	male	ECOG 1	no SCC	Adenocarcinoma
41	negative	positive	67,4	male	ECOG 1	no SCC	Adenocarcinoma
42	negative	positive	64,5	male	ECOG 1	no SCC	Adenocarcinoma
43	negative	positive	48,4	female	ECOG 1	no SCC	Adenocarcinoma
44	negative	positive	43,6	female	ECOG 1	no SCC	Adenocarcinoma
45	positive	negative	60,7	male	ECOG 1	no SCC	Adenocarcinoma
46	positive	negative	67,9	male	ECOG 1	no SCC	Adenocarcinoma

47	positive	negative	65,1	male	ECOG 1	no SCC	Adenocarcinoma
48	positive	negative	78,7	male	ECOG 1	no SCC	Adenocarcinoma
49	negative	negative	64,5	male	ECOG 1	no SCC	Adenocarcinoma
50	negative	negative	62,1	male	ECOG 1	no SCC	Adenocarcinoma
51	negative	negative	68,6	male	ECOG 1	no SCC	Adenocarcinoma
52	negative	negative	64,3	male	ECOG 1	no SCC	Adenocarcinoma
53	negative	negative	71	male	ECOG 1	no SCC	Adenocarcinoma
54	negative	negative	73	male	ECOG 1	no SCC	Adenocarcinoma
55	negative	negative	69,7	male	ECOG 1	no SCC	Adenocarcinoma
56	negative	negative	61,7	male	ECOG 1	no SCC	Adenocarcinoma
57	negative	negative	64,4	male	ECOG 1	no SCC	Adenocarcinoma
58	negative	negative	60,4	female	ECOG 1	no SCC	Adenocarcinoma
59	negative	negative	44,3	male	ECOG 1	no SCC	Adenocarcinoma
60	negative	negative	71,1	male	ECOG 1	no SCC	Adenocarcinoma
61	negative	negative	61,5	male	ECOG 1	no SCC	Adenocarcinoma
62	negative	negative	48,2	female	ECOG 1	no SCC	Adenocarcinoma
63	negative	negative	69,2	male	ECOG 1	no SCC	Adenocarcinoma
64	negative	negative	40,6	male	ECOG 1	no SCC	Adenocarcinoma
65	negative	negative	74,4	male	ECOG 1	no SCC	Adenocarcinoma
66	negative	negative	64,6	male	ECOG 1	no SCC	Adenocarcinoma
67	negative	negative	75,3	male	ECOG 1	no SCC	Adenocarcinoma
68	negative	negative	66,1	female	ECOG 1	no SCC	Adenocarcinoma
69	negative	negative	54,6	female	ECOG 1	no SCC	Adenocarcinoma
70	negative	negative	72,5	male	ECOG 1	no SCC	Adenocarcinoma
71	negative	negative	52,4	male	ECOG 1	no SCC	Adenocarcinoma
72	negative	negative	57,3	female	ECOG 1	no SCC	Adenocarcinoma
73	negative	negative	65,7	male	ECOG 1	no SCC	Adenocarcinoma
74	negative	negative	62,3	male	ECOG 1	no SCC	Adenocarcinoma
75	negative	negative	70,4	female	ECOG 1	no SCC	Adenocarcinoma
76	negative	negative	64,2	male	ECOG 1	no SCC	Adenocarcinoma
77	negative	negative	61,7	male	ECOG 1	no SCC	Adenocarcinoma
78	negative	negative	58,7	male	ECOG 1	no SCC	Adenocarcinoma
79	negative	negative	78,1	male	ECOG 1	no SCC	Adenocarcinoma
80	negative	negative	77,6	female	ECOG 1	no SCC	Adenocarcinoma
81	negative	negative	72,8	female	ECOG 1	no SCC	Adenocarcinoma
82	negative	negative	67,6	female	ECOG 1	no SCC	Adenocarcinoma
83	negative	negative	69,7	female	ECOG 1	no SCC	Adenocarcinoma
84	negative	negative	63,4	female	ECOG 1	no SCC	Adenocarcinoma
85	negative	negative	75,2	male	ECOG 1	no SCC	Adenocarcinoma
86	negative	negative	66	male	ECOG 1	no SCC	Adenocarcinoma
87	negative	negative	67,6	male	ECOG 1	no SCC	Adenocarcinoma
88	negative	negative	52,3	male	ECOG 1	no SCC	Adenocarcinoma
89	negative	negative	64,1	male	ECOG 1	no SCC	Adenocarcinoma
90	negative	negative	66,7	female	ECOG 1	no SCC	Adenocarcinoma
91	negative	negative	71,9	male	ECOG 1	no SCC	Adenocarcinoma
92	negative	negative	72,1	female	ECOG 1	no SCC	Adenocarcinoma
93	negative	negative	72,4	female	ECOG 1	no SCC	Adenocarcinoma
94	negative	negative	70,6	female	ECOG 1	no SCC	Adenocarcinoma
95	negative	negative	72,2	male	ECOG 1	no SCC	Adenocarcinoma
96	negative	negative	76,1	female	ECOG 1	no SCC	Adenocarcinoma

97	negative	negative	65	male	ECOG 1	no SCC	Adenocarcinoma
98	negative	positive	65,1	male	ECOG 1	Squamous cell carcinoma	no ACA
99	negative	positive	62,1	male	ECOG 1	Squamous cell carcinoma	no ACA
100	positive	negative	59,3	male	ECOG 1	Squamous cell carcinoma	no ACA
101	positive	negative	54,2	male	ECOG 1	Squamous cell carcinoma	no ACA
102	positive	negative	65,2	female	ECOG 1	Squamous cell carcinoma	no ACA
103	positive	negative	62,3	male	ECOG 1	Squamous cell carcinoma	no ACA
104	positive	negative	78,6	male	ECOG 1	Squamous cell carcinoma	no ACA
105	positive	negative	58,7	male	ECOG 1	Squamous cell carcinoma	no ACA
106	positive	negative	67,5	male	ECOG 1	Squamous cell carcinoma	no ACA
107	positive	negative	58,2	male	ECOG 1	Squamous cell carcinoma	no ACA
108	positive	negative	74,2	male	ECOG 1	Squamous cell carcinoma	no ACA
109	positive	negative	51,8	male	ECOG 1	Squamous cell carcinoma	no ACA
110	positive	negative	76,4	male	ECOG 1	Squamous cell carcinoma	no ACA
111	negative	negative	64,5	male	ECOG 1	Squamous cell carcinoma	no ACA
112	negative	negative	58,5	male	ECOG 1	Squamous cell carcinoma	no ACA
113	negative	negative	68,9	male	ECOG 1	Squamous cell carcinoma	no ACA
114	negative	negative	65,7	male	ECOG 1	Squamous cell carcinoma	no ACA
115	negative	negative	70,7	male	ECOG 1	Squamous cell carcinoma	no ACA
116	negative	negative	72,3	male	ECOG 1	Squamous cell carcinoma	no ACA
117	negative	negative	74,8	male	ECOG 1	Squamous cell carcinoma	no ACA
118	negative	negative	63,2	male	ECOG 1	Squamous cell carcinoma	no ACA
119	negative	negative	60,2	male	ECOG 1	Squamous cell carcinoma	no ACA
120	negative	negative	52,7	male	ECOG 1	Squamous cell carcinoma	no ACA
121	negative	negative	75,1	male	ECOG 1	Squamous cell carcinoma	no ACA
122	negative	negative	75,3	male	ECOG 1	Squamous cell carcinoma	no ACA
123	negative	negative	75,8	male	ECOG 1	Squamous cell carcinoma	no ACA
124	negative	negative	62,3	male	ECOG 1	Squamous cell carcinoma	no ACA
125	negative	negative	57,3	male	ECOG 1	Squamous cell carcinoma	no ACA
126	negative	negative	68,2	male	ECOG 1	Squamous cell carcinoma	no ACA
127	negative	negative	58,8	male	ECOG 1	Squamous cell carcinoma	no ACA
128	negative	negative	64,1	male	ECOG 1	Squamous cell carcinoma	no ACA
129	negative	negative	81,7	male	ECOG 1	Squamous cell carcinoma	no ACA
130	negative	negative	58,4	female	ECOG 1	Squamous cell carcinoma	no ACA
131	negative	negative	71,9	female	ECOG 1	Squamous cell carcinoma	no ACA
132	negative	negative	72,8	male	ECOG 1	Squamous cell carcinoma	no ACA
133	negative	negative	64,7	male	ECOG 1	Squamous cell carcinoma	no ACA
134	negative	negative	65	male	ECOG 1	Squamous cell carcinoma	no ACA
135	negative	negative	49,3	male	ECOG 1	Squamous cell carcinoma	no ACA
136	negative	negative	70,7	male	ECOG 1	Squamous cell carcinoma	no ACA
137	negative	negative	72,2	male	ECOG 1	Squamous cell carcinoma	no ACA
138	negative	negative	63,4	male	ECOG 1	Squamous cell carcinoma	no ACA
139	negative	negative	81,5	female	ECOG 1	Squamous cell carcinoma	no ACA
140	negative	negative	49	male	ECOG 1	Squamous cell carcinoma	no ACA
141	negative	negative	74,7	male	ECOG 1	Squamous cell carcinoma	no ACA
142	negative	negative	80,3	female	ECOG 1	Squamous cell carcinoma	no ACA
143	negative	negative	79,6	male	ECOG 1	Squamous cell carcinoma	no ACA
144	negative	negative	63,6	male	ECOG 1	Squamous cell carcinoma	no ACA
145	negative	negative	69,7	male	ECOG 1	Squamous cell carcinoma	no ACA
146	negative	negative	77	male	ECOG 1	Squamous cell carcinoma	no ACA

147	negative	negative	70,2	male	ECOG 1	Squamous cell carcinoma	no ACA
148	negative	negative	45,4	male	ECOG 1	Squamous cell carcinoma	no ACA
149	negative	negative	64,6	male	ECOG 1	Squamous cell carcinoma	no ACA
150	negative	negative	71,1	male	ECOG 1	Squamous cell carcinoma	no ACA
151	negative	negative	78,8	male	ECOG 1	Squamous cell carcinoma	no ACA
152	negative	negative	47,2	male	ECOG 1	Squamous cell carcinoma	no ACA
153	negative	negative	68,1	male	ECOG 1	Squamous cell carcinoma	no ACA
154	negative	negative	64,6	male	ECOG 1	Squamous cell carcinoma	no ACA
155	negative	negative	68,5	female	ECOG 1	Squamous cell carcinoma	no ACA
156	negative	negative	77,4	female	ECOG 1	Squamous cell carcinoma	no ACA
157	negative	negative	64,5	male	ECOG 1	Squamous cell carcinoma	no ACA
158	negative	negative	66,7	male	ECOG 1	Squamous cell carcinoma	no ACA
159	negative	negative	66	male	ECOG 1	Squamous cell carcinoma	no ACA
160	negative	negative	61,9	male	ECOG 1	Squamous cell carcinoma	no ACA
161	negative	negative	73,8	female	ECOG 1	Squamous cell carcinoma	no ACA
162	negative	negative	64,8	male	ECOG 1	Squamous cell carcinoma	no ACA
163	negative	negative	62,6	female	ECOG 1	Squamous cell carcinoma	no ACA
164	negative	negative	78,8	male	ECOG 1	Squamous cell carcinoma	no ACA
165	negative	negative	64,8	male	ECOG 1	Squamous cell carcinoma	no ACA
166	negative	negative	69,9	male	ECOG 1	Squamous cell carcinoma	no ACA
167	negative	negative	64,7	male	ECOG 1	Squamous cell carcinoma	no ACA
168	negative	negative	82,8	male	ECOG 1	no SCC	no ACA
169	negative	negative	64,5	male	ECOG 1	no SCC	no ACA
170	negative	negative	58,8	female	ECOG 1	no SCC	no ACA
171	negative	negative	68,3	male	ECOG 1	no SCC	no ACA
172	negative	negative	52,3	male	ECOG 1	no SCC	no ACA
173	negative	negative	63,2	male	ECOG 1	no SCC	no ACA
174	negative	positive	76,4	female	ECOG 1	no SCC	Adenocarcinoma
175	negative	positive	64,4	male	ECOG 1	no SCC	Adenocarcinoma
176	positive	negative	66,6	male	ECOG 1	no SCC	Adenocarcinoma
177	positive	negative	72,6	male	ECOG 1	no SCC	Adenocarcinoma
178	negative	negative	55,6	male	ECOG 1	no SCC	Adenocarcinoma
179	negative	negative	61,5	female	ECOG 1	no SCC	Adenocarcinoma
180	negative	negative	67,7	male	ECOG 1	no SCC	Adenocarcinoma
181	negative	negative	62,3	male	ECOG 1	no SCC	Adenocarcinoma
182	negative	negative	67,8	male	ECOG 1	no SCC	Adenocarcinoma
183	negative	negative	66,2	male	ECOG 1	no SCC	Adenocarcinoma
184	negative	positive	62,4	male	ECOG 1	Squamous cell carcinoma	no ACA
185	positive	negative	63,9	male	ECOG 1	Squamous cell carcinoma	no ACA
186	negative	negative	58,5	male	ECOG 1	Squamous cell carcinoma	no ACA
187	negative	negative	71,9	female	ECOG 1	Squamous cell carcinoma	no ACA
188	negative	negative	84,4	male	ECOG 1	Squamous cell carcinoma	no ACA
189	negative	negative	66,8	male	ECOG 1	Squamous cell carcinoma	no ACA
190	negative	negative	69,2	male	ECOG 1	Squamous cell carcinoma	no ACA
191	negative	negative	70,3	male	ECOG 1	Squamous cell carcinoma	no ACA
192	negative	negative	63,4	male	ECOG 1	Squamous cell carcinoma	no ACA
193	negative	negative	74,4	male	ECOG 1	Squamous cell carcinoma	no ACA
194	negative	negative	69,1	male	ECOG 1	Squamous cell carcinoma	no ACA
195	negative	negative	64	male	ECOG 1	Squamous cell carcinoma	no ACA
196	negative	negative	70,1	male	ECOG 1	Squamous cell carcinoma	no ACA

197	negative	negative	78,2	male	ECOG 1	Squamous cell carcinoma	no ACA
198	negative	negative	59,3	male	ECOG 1	Squamous cell carcinoma	no ACA
199	negative	negative	66	male	ECOG 1	Squamous cell carcinoma	no ACA
200	negative	negative	66,1	male	ECOG 1	Squamous cell carcinoma	no ACA
201	negative	negative	72,3	male	ECOG 1	Squamous cell carcinoma	no ACA
202	positive	negative	52	female	ECOG 1	no SCC	no ACA
203	positive	negative	66,6	male	ECOG 1	no SCC	no ACA
204	negative	negative	70	female	ECOG 1	no SCC	no ACA
205	negative	negative	64,3	male	ECOG 1	no SCC	no ACA
206	negative	negative	69,7	male	ECOG 1	no SCC	Adenocarcinoma
207	negative	negative	77,9	male	ECOG 1	no SCC	Adenocarcinoma
208	negative	negative	70,9	female	ECOG 1	Squamous cell carcinoma	no ACA
209	negative	negative	67,8	male	ECOG 1	Squamous cell carcinoma	no ACA
210	negative	negative	60,3	male	ECOG 1	Squamous cell carcinoma	no ACA
211	positive	negative	62,3	male	ECOG 0	no SCC	no ACA
212	positive	negative	71,2	male	ECOG 0	no SCC	no ACA
213	negative	negative	42,9	male	ECOG 0	no SCC	no ACA
214	negative	negative	61	male	ECOG 0	no SCC	no ACA
215	positive	positive	83,2	female	ECOG 0	no SCC	Adenocarcinoma
216	positive	positive	59,5	male	ECOG 0	no SCC	Adenocarcinoma
217	negative	positive	49	male	ECOG 0	no SCC	Adenocarcinoma
218	negative	negative	75,6	female	ECOG 0	no SCC	Adenocarcinoma
219	negative	negative	77,4	male	ECOG 0	no SCC	Adenocarcinoma
220	negative	negative	65	female	ECOG 0	no SCC	Adenocarcinoma
221	negative	negative	42,9	female	ECOG 0	no SCC	Adenocarcinoma
222	negative	negative	77,2	female	ECOG 0	no SCC	Adenocarcinoma
223	negative	negative	62,9	male	ECOG 0	no SCC	Adenocarcinoma
224	negative	negative	52,8	female	ECOG 0	no SCC	Adenocarcinoma
225	negative	negative	58,3	male	ECOG 0	no SCC	Adenocarcinoma
226	negative	negative	49,1	female	ECOG 0	no SCC	Adenocarcinoma
227	negative	negative	53	male	ECOG 0	no SCC	Adenocarcinoma
228	negative	negative	58,9	male	ECOG 0	no SCC	Adenocarcinoma
229	negative	negative	76,7	female	ECOG 0	no SCC	Adenocarcinoma
230	negative	negative	73,3	male	ECOG 0	no SCC	Adenocarcinoma
231	negative	negative	66,9	male	ECOG 0	no SCC	Adenocarcinoma
232	positive	negative	54,6	male	ECOG 0	Squamous cell carcinoma	no ACA
233	negative	negative	70,2	male	ECOG 0	Squamous cell carcinoma	no ACA
234	negative	negative	74,4	male	ECOG 0	Squamous cell carcinoma	no ACA
235	negative	negative	64,3	male	ECOG 0	Squamous cell carcinoma	no ACA
236	negative	negative	70,2	male	ECOG 0	Squamous cell carcinoma	no ACA
237	negative	negative	69,5	male	ECOG 0	Squamous cell carcinoma	no ACA
238	negative	negative	65,2	male	ECOG 0	Squamous cell carcinoma	no ACA
239	negative	negative	65,3	male	ECOG 0	Squamous cell carcinoma	no ACA
240	positive	positive	53,1	female	ECOG 0	no SCC	Adenocarcinoma
241	negative	positive	67,8	female	ECOG 0	no SCC	Adenocarcinoma
242	positive	negative	73,8	female	ECOG 0	no SCC	Adenocarcinoma
243	positive	negative	59,6	female	ECOG 0	no SCC	Adenocarcinoma
244	positive	negative	71,5	female	ECOG 0	no SCC	Adenocarcinoma
245	negative	negative	44,6	male	ECOG 0	no SCC	Adenocarcinoma
246	negative	negative	57,8	female	ECOG 0	no SCC	Adenocarcinoma

247	positive	negative	63,7	male	ECOG 0	Squamous cell carcinoma	no ACA
248	positive	negative	68,5	male	ECOG 0	Squamous cell carcinoma	no ACA
249	negative	negative	63,6	male	ECOG 0	Squamous cell carcinoma	no ACA
250	negative	negative	75,1	male	ECOG 0	Squamous cell carcinoma	no ACA
251	negative	negative	53,8	male	ECOG 0	Squamous cell carcinoma	no ACA
252	negative	negative	58,8	male	ECOG 0	Squamous cell carcinoma	no ACA
253	negative	negative	75,6	male	ECOG 0	Squamous cell carcinoma	no ACA
254	negative	negative	59,2	male	ECOG 0	Squamous cell carcinoma	no ACA
255	negative	negative	73,9	male	ECOG 0	Squamous cell carcinoma	no ACA
256	negative	negative	59,8	male	ECOG 0	Squamous cell carcinoma	no ACA
257	positive	negative	49,1	male	ECOG 0	no SCC	no ACA
258	negative	negative	74,5	male	ECOG 0	no SCC	Adenocarcinoma
259	negative	negative		male		no SCC	no ACA
260	negative	negative	66,7	male		no SCC	Adenocarcinoma
261	negative	negative	64,4	male		no SCC	Adenocarcinoma
262	positive	negative	60,9	female		Squamous cell carcinoma	no ACA
263	positive	negative	57,2	male		Squamous cell carcinoma	no ACA
264	negative	negative	64,7	male		Squamous cell carcinoma	no ACA
265	negative	negative	67,2	male		Squamous cell carcinoma	no ACA
266	negative	negative	66	female		no SCC	Adenocarcinoma
267	negative	negative	72,4	male		Squamous cell carcinoma	no ACA
268	positive	negative	63,7	female		no SCC	Adenocarcinoma
269	negative	negative	73,5	male		no SCC	Adenocarcinoma
270	negative	negative	56,4	female		no SCC	Adenocarcinoma

Raw data: NSCLC stuyc collective; CD13 expression

#	Large Cell Carcinoma	pN	Stage	Grading	Survival status	Overall Survival
1	Large Cell Carcinoma	0	Stage 1	G3	dead	1460
2	no LCC	0	Stage 1	G3	dead	854
3	no LCC	0	Stage 1	G3	dead	418
4	no LCC	0	Stage 1	G3	dead	2874
5	no LCC	0	Stage 1	G3	alive	1346
6	no LCC	0	Stage 1	G3	dead	2879
7	no LCC	0	Stage 1	G2	alive	3360
8	no LCC	0	Stage 1	G3	dead	801
9	no LCC	0	Stage 1	G3	dead	169
10	no LCC	1	Stage 3	G3	dead	79
11	no LCC	1	Stage 2	G3	dead	347
12	no LCC	2	Stage 3	G3	dead	227
13	no LCC	2	Stage 3	G3	dead	360
14	no LCC	2	Stage 3	G3	dead	2199
15	Large Cell Carcinoma	0	Stage 2	G4	dead	62
16	Large Cell Carcinoma	0	Stage 1	G4	alive	963
17	Large Cell Carcinoma	0	Stage 1	G3	dead	2019
18	Large Cell Carcinoma	0	Stage 1		dead	2083
19	Large Cell Carcinoma	0	Stage 1	G4	dead	684
20	Large Cell Carcinoma	0	Stage 3	G4	alive	852
21	Large Cell Carcinoma	0	Stage 1	G3	dead	1223
22	Large Cell Carcinoma	0	Stage 1	G4	dead	2053
23	Large Cell Carcinoma	0	Stage 1	G4	alive	1951
24	Large Cell Carcinoma	0	Stage 1	G4	dead	306
25	Large Cell Carcinoma	0	Stage 1	G4	dead	226
26	Large Cell Carcinoma	0	Stage 1	G4	dead	151
27	Large Cell Carcinoma	0	Stage 2	G4	dead	1257
28	Large Cell Carcinoma	0	Stage 1	G4	alive	3202
29	Large Cell Carcinoma	0	Stage 1	G4	alive	3025
30	Large Cell Carcinoma	0	Stage 1	G4	alive	3748
31	Large Cell Carcinoma	0	Stage 1	G4	dead	937
32	Large Cell Carcinoma	0	Stage 1	G4	dead	51
33	Large Cell Carcinoma	0	Stage 1	G4	dead	233
34	Large Cell Carcinoma	0	Stage 1	G4	dead	264
35	Large Cell Carcinoma	0	Stage 1	G4	dead	513
36	Large Cell Carcinoma	0	Stage 1		dead	507
37	Large Cell Carcinoma	0	Stage 1	G3	dead	342
38	no LCC	0	Stage 1	G3	dead	1460
39	no LCC	0	Stage 1	G3	alive	2155
40	no LCC	0	Stage 1	G2	alive	2128
41	no LCC	0	Stage 1	G2	dead	1056
42	no LCC	0	Stage 1	G2	dead	941
43	no LCC	0	Stage 1	G2	alive	1040
44	no LCC	0	Stage 1	G2	dead	2727
45	no LCC	0	Stage 1	G3	dead	1963
46	no LCC	0	Stage 1	G3	dead	2096

47	no LCC	0	Stage 1	G3	dead	688
48	no LCC	0	Stage 1	G3	dead	1324
49	no LCC	0	Stage 1	G3	alive	2644
50	no LCC	0	Stage 1	G2	alive	1982
51	no LCC	0	Stage 1	G2	alive	2394
52	no LCC	0	Stage 1	G2	alive	2521
53	no LCC	0	Stage 1	G3	dead	403
54	no LCC	0	Stage 1	G2	dead	2207
55	no LCC	0	Stage 3	G2	dead	923
56	no LCC	0	Stage 1	G1	alive	2905
57	no LCC	0	Stage 1	G3	alive	2888
58	no LCC	0	Stage 1	G2	dead	234
59	no LCC	0	Stage 1	G3	alive	2535
60	no LCC	0	Stage 1	G2	alive	3902
61	no LCC	0	Stage 1	G3	dead	157
62	no LCC	0	Stage 1	G3	alive	3934
63	no LCC	0	Stage 1	G3	alive	3101
64	no LCC	0	Stage 1	G3	alive	2681
65	no LCC	0	Stage 1	G3	alive	2518
66	no LCC	0	Stage 1	G3	alive	936
67	no LCC	0	Stage 1	G2	alive	1173
68	no LCC	0	Stage 1	G3	alive	3137
69	no LCC	0	Stage 1	G2	alive	3051
70	no LCC	0	Stage 1	G2	dead	1391
71	no LCC	0	Stage 1	G3	alive	2744
72	no LCC	0	Stage 1	G2	alive	2582
73	no LCC	0	Stage 1	G2	dead	1930
74	no LCC	0	Stage 1	G2	alive	2464
75	no LCC	0	Stage 1	G3	alive	2590
76	no LCC	0	Stage 1	G2	alive	2562
77	no LCC	0	Stage 1	G2	alive	2653
78	no LCC	0	Stage 1	G2	alive	2748
79	no LCC	0	Stage 1	G2	alive	2118
80	no LCC	0	Stage 1	G2	alive	2161
81	no LCC	0	Stage 1	G2	dead	185
82	no LCC	0	Stage 1	G2	alive	1138
83	no LCC	0	Stage 1	G2	alive	464
84	no LCC	0	Stage 1	G3	dead	3596
85	no LCC	0	Stage 1	G2	dead	246
86	no LCC	0	Stage 3	G3	dead	647
87	no LCC	0	Stage 1	G2	dead	1156
88	no LCC	0	Stage 1	G1	dead	4002
89	no LCC	0	Stage 1	G3	alive	2841
90	no LCC	0	Stage 1	G2	alive	2926
91	no LCC	0	Stage 1	G1	dead	2040
92	no LCC	0	Stage 3	G2	dead	1321
93	no LCC	0	Stage 1	G3	dead	225
94	no LCC	0	Stage 1	G3	alive	2911
95	no LCC	0	Stage 1	G3	dead	1540
96	no LCC	0	Stage 1	G2	alive	3387

97	no LCC	0	Stage 1	G1	dead	1256
98	no LCC	0	Stage 1	G2	alive	3715
99	no LCC	0	Stage 1	G3	dead	3120
100	no LCC	0	Stage 1	G2	alive	2173
101	no LCC	0	Stage 2	G3	alive	2414
102	no LCC	0	Stage 1	G3	alive	1567
103	no LCC	0	Stage 1	G3	alive	2485
104	no LCC	0	Stage 1	G3	dead	1662
105	no LCC	0	Stage 1	G2	alive	1482
106	no LCC	0	Stage 1	G2	dead	522
107	no LCC	0	Stage 1	G3	alive	3106
108	no LCC	0	Stage 2	G3	dead	470
109	no LCC	0	Stage 1	G2	alive	2808
110	no LCC	0	Stage 1	G2	dead	299
111	no LCC	0	Stage 1	G3	dead	876
112	no LCC	0	Stage 1	G3	dead	293
113	no LCC	0	Stage 1	G3	dead	499
114	no LCC	0	Stage 1	G3	dead	1390
115	no LCC	0	Stage 1	G2	dead	903
116	no LCC	0	Stage 1	G3	alive	2596
117	no LCC	0	Stage 1	G3	dead	503
118	no LCC	0	Stage 1	G3	alive	2484
119	no LCC	0	Stage 1	G3	alive	3269
120	no LCC	0	Stage 1	G3		
121	no LCC	0	Stage 1	G3	alive	2747
122	no LCC	0	Stage 1	G2	dead	840
123	no LCC	0	Stage 1	G3	dead	771
124	no LCC	0	Stage 1	G2	dead	91
125	no LCC	0	Stage 1	G2	dead	844
126	no LCC	0	Stage 1	G3	alive	1244
127	no LCC	0	Stage 1	G3	alive	2828
128	no LCC	0	Stage 1	G2	dead	2135
129	no LCC	0	Stage 1	G3	dead	1121
130	no LCC	0	Stage 1	G3	dead	137
131	no LCC	0	Stage 1	G3	dead	2141
132	no LCC	0	Stage 1	G3	dead	378
133	no LCC	0	Stage 1	G3	alive	2950
134	no LCC	0	Stage 1	G3	alive	2717
135	no LCC	0	Stage 1	G3	dead	1940
136	no LCC	0	Stage 1	G3	alive	1690
137	no LCC	0	Stage 1	G2	dead	1934
138	no LCC	0	Stage 3	G3	dead	382
139	no LCC	0	Stage 1	G3	alive	2558
140	no LCC	0	Stage 1	G3	dead	787
141	no LCC	0	Stage 1	G3	dead	669
142	no LCC	0	Stage 2	G2	dead	1076
143	no LCC	0	Stage 1	G3	dead	372
144	no LCC	0	Stage 1	G2		
145	no LCC	0	Stage 1	G3	alive	1791
146	no LCC	0	Stage 1	G3	dead	2873

147	no LCC	0	Stage 1	G2	dead	1599
148	no LCC	0	Stage 1	G3	alive	2349
149	no LCC	0	Stage 1	G2	dead	2217
150	no LCC	0	Stage 1	G2	alive	2574
151	no LCC	0	Stage 1	G2	dead	765
152	no LCC	0	Stage 1	G2		
153	no LCC	0	Stage 1	G4	dead	287
154	no LCC	0	Stage 2	G2	dead	136
155	no LCC	0	Stage 1	G3	alive	2656
156	no LCC	0	Stage 2	G3	dead	649
157	no LCC	0	Stage 1	G3	dead	785
158	no LCC	0	Stage 1	G2	alive	3074
159	no LCC	0	Stage 1	G3	dead	1092
160	no LCC	0	Stage 1	G3		
161	no LCC	0	Stage 1	G3	dead	1054
162	no LCC	0	Stage 1	G3	dead	685
163	no LCC	0	Stage 1	G3	dead	286
164	no LCC	0	Stage 2	G3	dead	934
165	no LCC	0	Stage 1	G3	dead	1592
166	no LCC	0	Stage 1	G3	dead	1031
167	no LCC	0	Stage 1	G2	dead	1231
168	Large Cell Carcinoma	1	Stage 2	G4	dead	713
169	Large Cell Carcinoma	1	Stage 2	G4	dead	281
170	Large Cell Carcinoma	1	Stage 2	G3	dead	1950
171	Large Cell Carcinoma	1	Stage 2		dead	434
172	Large Cell Carcinoma	1	Stage 2	G4	alive	4194
173	Large Cell Carcinoma	1	Stage 2	G4	dead	249
174	no LCC	1	Stage 2	G2	dead	756
175	no LCC	1	Stage 2	G2	dead	608
176	no LCC	1	Stage 2	G3	dead	137
177	no LCC	1	Stage 2	G2	alive	2232
178	no LCC	1	Stage 2	G2	dead	569
179	no LCC	1	Stage 2	G3	alive	
180	no LCC	1	Stage 2	G3	dead	337
181	no LCC	1	Stage 2	G3	alive	2775
182	no LCC	1	Stage 2	G2	dead	900
183	no LCC	1	Stage 2	G3	dead	2116
184	no LCC	1	Stage 3	G3	dead	478
185	no LCC	1	Stage 2	G2	dead	128
186	no LCC	1	Stage 2	G3	dead	311
187	no LCC	1	Stage 2	G2	dead	1657
188	no LCC	1	Stage 2	G3	dead	882
189	no LCC	1	Stage 2	G3	dead	226
190	no LCC	1	Stage 2	G3	alive	2897
191	no LCC	1	Stage 2	G3	alive	2473
192	no LCC	1	Stage 2	G3	alive	1118
193	no LCC	1	Stage 2	G3	dead	1322
194	no LCC	1	Stage 2	G3	dead	1241
195	no LCC	1	Stage 2	G3	dead	156
196	no LCC	1	Stage 3	G3	dead	920

197	no LCC	1	Stage 2	G2	dead	1068
198	no LCC	1	Stage 3	G2	alive	4025
199	no LCC	1	Stage 3	G3	alive	811
200	no LCC	1	Stage 2	G2	dead	40
201	no LCC	1	Stage 2	G3	alive	1495
202	Large Cell Carcinoma	2	Stage 3	G4	dead	80
203	Large Cell Carcinoma	2	Stage 3	G4	dead	277
204	Large Cell Carcinoma	2	Stage 3	G4	dead	511
205	Large Cell Carcinoma	2	Stage 3	G4	dead	2498
206	no LCC	2	Stage 3	G3	dead	1362
207	no LCC	2	Stage 3	G2	dead	1277
208	no LCC	2	Stage 3	G3	dead	509
209	no LCC	2	Stage 3	G3	dead	838
210	no LCC	2	Stage 3	G2	dead	1055
211	Large Cell Carcinoma	0	Stage 1	G4	dead	540
212	Large Cell Carcinoma	0	Stage 1	G3	alive	3422
213	Large Cell Carcinoma	0	Stage 1	G2	alive	3571
214	Large Cell Carcinoma	0	Stage 1	G4	dead	1742
215	no LCC	0	Stage 1	G2	alive	2497
216	no LCC	0	Stage 1	G1	dead	2419
217	no LCC	0	Stage 1	G2	dead	610
218	no LCC	0	Stage 1	G3	dead	353
219	no LCC	0	Stage 1	G3	dead	460
220	no LCC	0	Stage 1	G2	alive	2770
221	no LCC	0	Stage 1	G2	dead	1033
222	no LCC	0	Stage 1	G2	dead	763
223	no LCC	0	Stage 1	G2	dead	2740
224	no LCC	0	Stage 1	G2	alive	2627
225	no LCC	0	Stage 1	G3	alive	3711
226	no LCC	0	Stage 1	G2	alive	3745
227	no LCC	0	Stage 1	G2	dead	311
228	no LCC	0	Stage 1	G2	dead	1194
229	no LCC	0	Stage 1	G2	alive	2587
230	no LCC	0	Stage 1	G1	dead	810
231	no LCC	0	Stage 2	G3	alive	2678
232	no LCC	0	Stage 1	G2	alive	2894
233	no LCC	0	Stage 1	G3	dead	527
234	no LCC	0	Stage 1	G3	dead	844
235	no LCC	0	Stage 1	G3	dead	2408
236	no LCC	0	Stage 1	G3	alive	3180
237	no LCC	0	Stage 1	G3	dead	1620
238	no LCC	0	Stage 1	G3	dead	1028
239	no LCC	0	Stage 1	G3	dead	2595
240	no LCC	1	Stage 2	G2	alive	3365
241	no LCC	1	Stage 2	G3	dead	1597
242	no LCC	1	Stage 2	G2	dead	2605
243	no LCC	1	Stage 2	G3	alive	2268
244	no LCC	1	Stage 2	G3	dead	667
245	no LCC	1	Stage 2	G3	dead	2612
246	no LCC	1	Stage 2	G2	dead	269

247	no LCC	1	Stage 2	G3	dead	884
248	no LCC	1	Stage 2	G3	alive	1059
249	no LCC	1	Stage 2	G3	dead	614
250	no LCC	1	Stage 2		dead	438
251	no LCC	1	Stage 2	G2	alive	2948
252	no LCC	1	Stage 2	G3	dead	802
253	no LCC	1	Stage 2	G3	dead	582
254	no LCC	1	Stage 2	G3	alive	4034
255	no LCC	1	Stage 2	G3	dead	1316
256	no LCC	1	Stage 2	G2	dead	776
257	Large Cell Carcinoma	2	Stage 3	G4	dead	180
258	no LCC	2	Stage 3	G2	dead	1205
259	Large Cell Carcinoma	0	Stage 1			
260	no LCC	0	Stage 1	G2	alive	1531
261	no LCC	0	Stage 1	G3	alive	1431
262	no LCC	0	Stage 1	G2	alive	1416
263	no LCC	0	Stage 1	G2	dead	1157
264	no LCC	0	Stage 1	G3	dead	28
265	no LCC	0	Stage 1	G2	dead	506
266	no LCC	1	Stage 2	G3	alive	1237
267	no LCC	1	Stage 2	G3	dead	197
268	no LCC	2	Stage 3	G2	dead	797
269	no LCC	2	Stage 3	G2	dead	1287
270	no LCC	2	Stage 3	G3	dead	157
